# The outcomes and quality of life of young patients undergoing adjuvant radiotherapy versus non-radiotherapy following surgery treating early FIGO stage cervical squamous cell cancer in southwestern China

**DOI:** 10.1038/s41598-020-66661-y

**Published:** 2020-06-12

**Authors:** Lingyun Yang, Jialing Yuan, Xi Zeng, Mingrong Xi, Hongjing Wang

**Affiliations:** 1 Department of Obstetrics and Gynecology, West China Second University Hospital, Chengdu, China; 2 Key Laboratory of Birth Defects and Related Diseases of Women and Children (Sichuan University), Ministry of Education, Chengdu, China

**Keywords:** Quality of life, Gynaecological cancer

## Abstract

Background: The incidence of cervical cancer in young women is rising, and squamous cell carcinoma makes up a great percentage of the histological types. The presence of aggressive pathologic risk factors following patients’ primary surgery may warrant the use of adjuvant radiotherapy. It is important to weigh up the risks and benefits of using adjuvant radiotherapy for each young patient so as to maximize their prognosis while minimizing the treatment-related morbidity. Methods: A retrospective study was performed. It consisted of 97 patients under 35 years old who were diagnosed with cervical squamous cell carcinoma and underwent treatment at West China Second University Hospital between December 2009 and January 2014. Five-year follow-up, prognostic risks, long-term radiation toxicity, female sexual function, and quality of life were investigated. Results: Adjuvant radiotherapy did improve the prognosis of young patients with lymph node metastases. However, there were few significant differences in progress-free survival and overall survival for the young patients without lymph node metastases following adjuvant radiotherapy. Besides, young patients who took radiotherapy exhibited greater intestinal dysfunction, more severe lower extremities edema, greater sexual dysfunction, and worse long-term quality of life. Conclusion: Young patients with early-stage cervical squamous cell carcinoma without lymph node metastases who have undergone the primary surgery should be counseled in detail before the decision to use adjuvant radiotherapy can be made. The counseling should emphasize not only the benefit that local recurrence rates can be reduced, but also the risks that treatment-related side effects could increase and lower QoL could occur.

## Introduction

Thanks to widespread screening and advanced medical treatment, the incidence and the mortality of cervical cancer have been reduced in developed countries^1^. However, in the rural developing regions of China, the morbidity of cervical cancer is still high due to the suboptimal medical conditions, making the cervical cancer a major health problem for women^2,3^. Recent studies showed that the incidence of cervical cancer in young women is rising and squamous cell carcinoma (SCC) still makes up a great percentage of the histological types^4,5^. To our best knowledge, the persistent infection of high-risk human papillomavirus (HPV) is identified as the most critical factor for the development of cervical cancer^6,7^. For young women, the squamous-columnar junction of cervix is more vulnerable to HPV infection if she has an early age of active intercourse.

Considering the quality of life (QoL), a primary surgery with radical hysterectomy or trachelectomy without bilateral salpingo-oophorectomy is usually performed for young women with early-stage cervical SCC. According to the National Comprehensive Cancer Network clinical practice guidelines of cervical cancer, if one or more aggressive pathologic risk factors are discoverd after the primary surgery, such as bulky tumor size, deep stromal invasion (DSI), lymph-vascular space invasion (LVSI), pelvic lymph nodes (LN) metastases, parametrial and surgical margin involvement, the use of adjuvant radiotherapy (RT) is warranted. However, it has been reported that using more than one treatments for cervical cancer leads to a potenital increase of related complications and side effects, such as diarrhea, bloody stool, urinary frequency, lower extremities edema, and sexual dysfuction^8,9^.

Taking into account long-term side effects, sexual function and QoL, adjuvant RT may be declined by young patients with cervical cancer. It is important to weigh up the risks and benefits of using adjuvant RT for each patient so as to maximize their prognosis while minimizing the treatment-related morbidity. Therefore, this study aims to make clear the impact of adjuvant RT on progress-free survival (PFS), overall survival (OS), treatment-related side effects, sexual function, and QoL for patients under 35 years old with early-stage cervical SCC following their primary surgery, compared with that of non-RT (NRT)..

## Patients and Methods

This study was performed at the department of gynecology and obstetrics of West China Second University Hospital, Chengdu, China. The follow-up and consent procedures were approved by the Sichuan University Medical Ethical Committee. We confirm that all research was performed in accordance with relevant regulations and informed consent was obtained from all participants.

A total of 191 patients under 35 years old had been diagnosed with cervical SCC and underwent treatment at our department between December 2009 and January 2014. 107 patients (10 patients lost to follow-up) were confirmed to present one or more pathologic risk factors after the primary surgery and were informed to take adjuvant RT. 23 patients out of them declined the following therapy. Consequently, patients were divided into two study groups: the RT and NRT groups. In the RT group, pelvic external beam radiotherapy (EBRT) with intensity-modulated radiation therapy (IMRT) technology was indicated. At the minimum, the radiation volume included upper 3 to 4 cm of the vaginal cuff, the parametria, and immediately adjacent nodal basins. For documented nodal metastasis, the common lilacs should be covered as well. For common iliac or para-aortic nodal involvement, the superior border of the radiation field was increased up to the level of the renal vessels. Vaginal brachytherapy was added as a useful boost for patients with positive pelvic nodes. Patients were treated with definitive EBRT to a dose of 45 to 50 Gy in standard fractionation. The fractionation schemes of brachytherapy included 5.5 Gy × 2 fractions dosed at 5 mm.

The clinical and pathologic characteristics of both study groups were examined. The follow-up included interval history, gynecological examination, and cervical smear test every 3 months for 1 year, every 6 months for another 2 years, and annually afterwards. Long-term radiation toxicity was documented based on the criteria of radiation morbidity scoring of the RT Oncology Group (RTOG) and the European Organization for Research and Treatment of Cancer (EORTC)^10^. The female sexual function index (FSFI) was used to assess sexual function in women, including desire, arousal, lubrication, orgasm, satisfaction, and pain. Higher scores indicate better sexual function^11^. The EORTC QLQ-C30 questionnaire was employed to assess the QoL. The standard score was calculated.

Statistical analysis was performed using SPSS software package. Pearson’s chi-square or Fisher exact’s test was implemented to compare the difference in proportions. The PFS and OS curves were constructed using the Kaplan-Meier method and were compared using the log-rank test. Univariate and multivariate analysis were performed using Cox’s multivariate regression model to identify meaningful prognostic factors. Differences in standard scores of female sexual function and QoL between two groups were evaluated using two-tailed Student’s t-test. P < 0.05 was considered to be statistically significant.

## Results

The clinical and pathologic characteristics of the two study groups are shown in Table 1. The proportion of FIGO stage II in the RT group was higher than that in the NRT group (45.9% vs 17.4%, P = 0.027). No significant differences in tumor differentiation, size, and pathologic risk factors were found between the two groups.

**Table 1:** Characteristics of 97 young patients with SCC in two study groups.

	n (%)	NRT	RT	P
		n = 23 (24.8%)	n = 74 (75.2%)	
Age, year				
Median		30	33	
Range		21–35	22–35	
Stage				0.027*
I	59 (60.8)	19 (82.6)	40 (54.1)	
II	38 (39.2)	4 (17.4)	34 (45.9)	
Differentiation				0.683
G1/G2	13 (13.4)	2 (8.7)	11 (14.9)	
G3	84 (86.6)	21 (91.3)	63 (85.1)	
Tumor size				0.911
<4 cm	60 (61.9)	14 (60.9)	46 (62.2)	
≥4 cm	37 (38.1)	9 (39.1)	28 (37.8)	
Stromal invasion				0.288
<1/2	18 (18.6)	6 (26.1)	12 (16.2)	
≥1/2	79 (81.4)	17 (73.9)	62 (83.8)	
LVSI				0.473
Negative	32 (33.0)	9 (39.1)	23 (31.1)	
Positive	65 (67.0)	14 (60.9)	51 (68.9)	
LN metastases				0.126
Negative	63 (64.9)	18 (78.3)	45 (60.8)	
Positive	34 (35.1)	5 (21.7))	29 (39.2)	

^*^P < 0.05.

**Figure 1:**
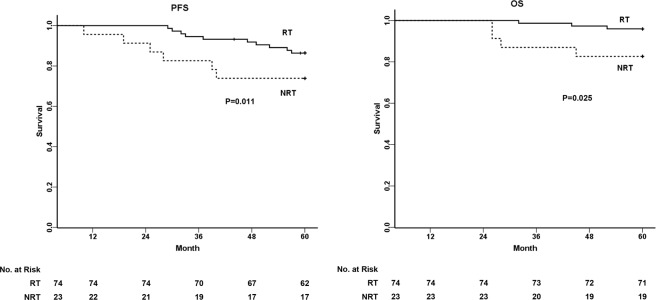
Survival analysis of young patients with SCC who were informed to take adjuvant radiotherapy. PFS and OS of 97 young patients are shown between two study groups: NRT and RT. The five-year PFS in NRT and RT groups were 74.0% vs. 86.4% (P = 0.011), and the five-year OS in NRT and RT groups were 82.6% vs. 95.9% (P = 0.025).

**Figure 2:**
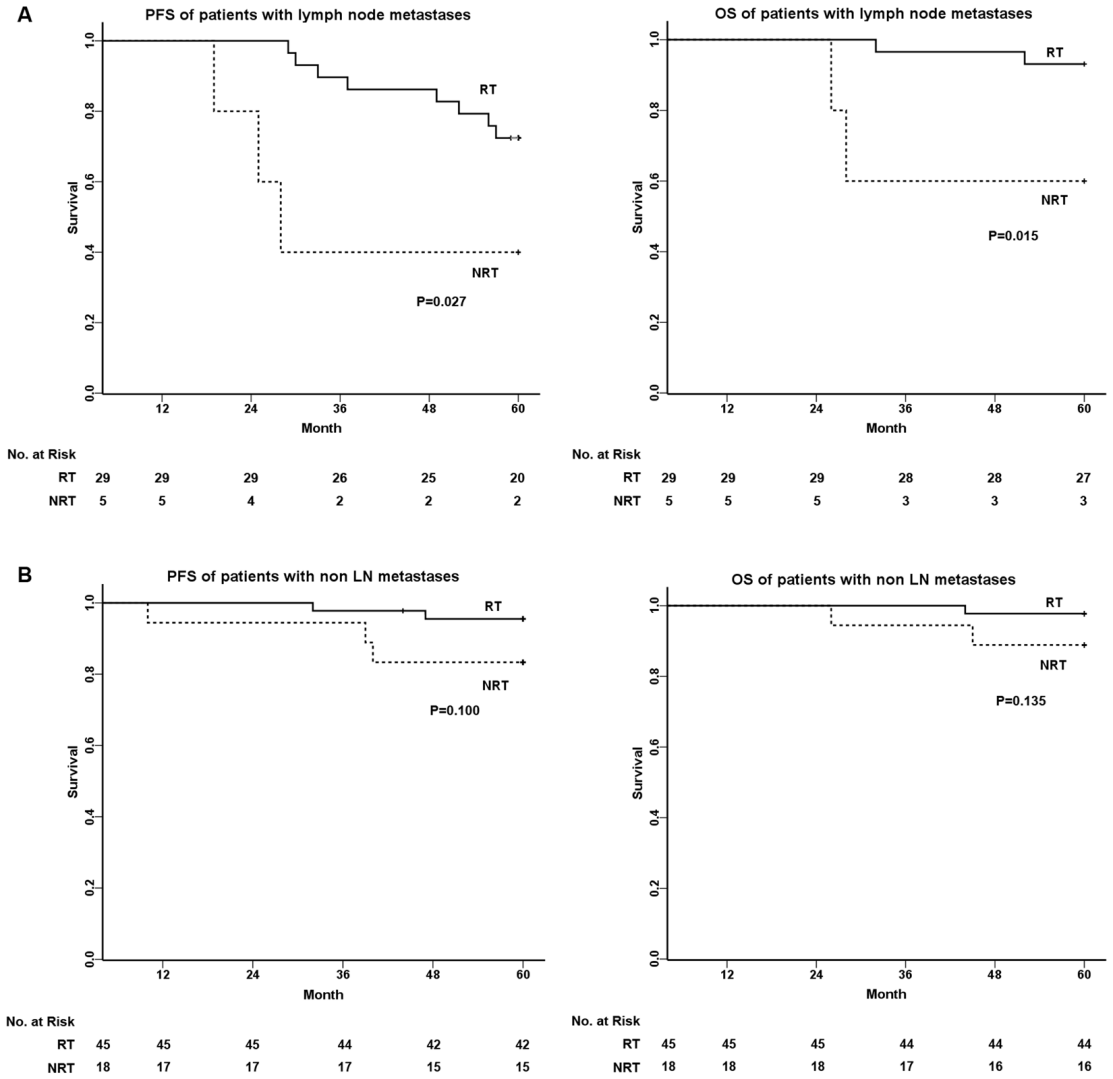
Survival analysis of young patients with SCC related to LN metastases between two study groups: NRT and RT. **(A)** For patients with LN metastases, the five-year PFS in NRT and RT groups were 40.0% vs. 72.4% (P = 0.027), and the five-year OS in NRT and RT groups were 60.0% vs. 93.1% (P = 0.015). **(B)** For patients with non-LN metastases, the five-year PFS in NRT and RT groups were 83.3% vs. 95.5% (P = 0.100), and the five-year OS in NRT and RT groups were 88.9% vs. 97.8% (P = 0.135).

The five-year PFS and OS of all 97 patients were 83.4% and 92.8%, respectively. Adjuvant RT was the significant prognostic factor of the poor PFS (NRT vs. RT = 74.0% vs. 86.4%, P = 0.011) and OS (NRT vs. RT = 82.6% vs. 95.9%, P = 0.025) in young patients with SCC (Fig. 1). Among prognostic factors, LN metastases and adjuvant RT were identified as independent prognostic factors for predicting PFS and OS using Cox’s multivariate regression analysis (Table 2). For patients with LN metastases (Fig. 2A), the five-year PFS and OS were 72.4% and 93.1% in the RT group, respectively, in contrast to 40.0% and 60.0% in the NRT group (P < 0.05). However, for patients with non-LN metastases (Fig. 2B), the five-year PFS and OS had few significant differences between both groups (P > 0.05).

**Table 2:** Prognostic factors of PFS and OS analyzed by Cox proportional hazard models for young patients with SCC.

Prognostic factors	PFS			OS		
	Hazard ratio	95%CI	P	Hazard ratio	95%CI	P
Bulky tumor	1.198	0.426–3.375	0.733	3.286	0.703–15.348	0.130
DSI	2.300	0.527–10.041	0.268	0.920	0.138–6.155	0.932
LVSI	1..451	0.464–4.539	0.522	4.264	0.505–35.962	0.183
LN metastases	11.029	2.742–44.358	0.001**	9.151	1.165–71.857	0.035*
Adjuvant RT	6.356	1.689–23.917	0.006**	12.260	1.646–91.329	0.014*

^*^P < 0.05.

**P < 0.01.

**Figure 3:**
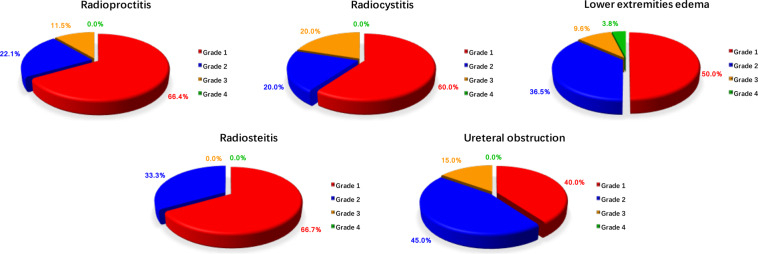
Long-term radiation toxicity of young patients with cervical SCC. The most common long-term radiation toxicity included radioproctitis, radiocystitis, radiosteitis, lower extremities edema in grade 1, and ureteral obstruction in grade 2.

The incidences of side effects associated with the two adjuvant treatment modalities are shown in Table 3. Compared to the NRT group, patients who had received adjuvant RT exhibited a significantly higher incidence of diarrhea (P = 0.018), bloody stool (P = 0.007), lower extremities edema (P = 0.033), and vaginal dryness (P = 0.000). As shown in Fig. 3, the most common long-term radiation toxicity included radioproctitis, radiocystitis, radiosteitis, lower extremities edema in grade 1, and ureteral obstruction in grade 2. Grade 4 toxicity of lower extremities edema in 2 of the 52 (3.8%) patients were documented.

**Table 3:** Comparison of long-term side effects between two study groups.

	n	NRT	RT	P
		n = 23 (24.8%)	n = 74 (75.2%)	
Fatigue	48	8 (34.8)	40 (54.1)	0.152
Abdominal pain	79	17 (73.9)	62 (83.8)	0.358
Diarrhea	51	7 (30.4)	44 (59.5)	0.018*
Constipation	65	16 (69.6)	49 (66.2)	1.000
Bloody stool	38	3 (13.0)	35 (47.3)	0.007**
Dysuria	23	3 (13.0)	20 (27.0)	0.273
Urinary incontinence	26	9 (39.1)	17 (23.0)	0.127
Urinary frequency	24	8 (34.8)	16 (21.6)	0.201
Lower extremities edema	49	7 (30.4)	42 (56.8)	0.033*
Vaginal discharge increasing	21	8 (34.8)	13 (17.6)	0.090
Vaginal dryness	52	4 (17.4)	48 (64.9)	0.000**
Lower back pain	32	4 (17.4)	28 (37.8)	0.117
Dermal flushing	27	3 (13.0)	24 (32.4)	0.122

^*^P < 0.05.

**P < 0.01.

Table 4 revealed that adjuvant RT-treated subjects exhibited significantly greater sexual dysfunction (21.63 ± 1.64 vs. 22.58 ± 1.34, P = 0.013). Comparing to the NRT group, patients undergoing adjuvant RT often presented difficulty in lubrication (3.34 ± 0.64 vs. 3.69 ± 0.39, P = 0.016), decrease in sexual satisfaction (3.50 ± 0.57 vs. 3.79 ± 0.47, P = 0.030), and increase in dyspareunia (3.43 ± 0.52 vs. 3.68 ± 0.40, P = 0.038).

**Table 4:** Standard mean scores of sexual dysfunctions based on FSFI between two study groups.

	NRT (n = 23,)	RT (n = 74)	P
Desire	3.86 ± 0.67	3.90 ± 0.69	0.812
Arousal	3.64 ± 0.65	3.59 ± 0.71	0.758
Lubrication	3.69 ± 0.39	3.34 ± 0.64	0.016*
Orgasm	3.91 ± 0.61	3.85 ± 0.73	0.752
Satisfaction	3.79 ± 0.47	3.50 ± 0.57	0.030*
Pain	3.68 ± 0.40	3.43 ± 0.52	0.038*
Full scale	22.58 ± 1.34	21.63 ± 1.64	0.013*

^*^P < 0.05.

There were significant statistical differences in the QoL issues, including physical function, emotional function, constipation, financial difficulties, and global health status between the two modalities (Table 5). The NRT group scored higher on the scale of global health status than the RT group (72.10 ± 14.78 vs. 64.30 ± 16.35, P = 0.044). In functional scales, physical function (NRT vs. RT = 71.30 ± 18.90 vs. 61.80 ± 19.28, P = 0.043) and emotional function (NRT vs. RT = 68.12 ± 16.98 vs. 59.12 ± 16.15, P = 0.023) had minimal differences. Meanwhile, in symptom scales/items, diarrhea (NRT vs. RT = 72.46 ± 23.89 vs. 52.70 ± 33.11, P = 0.009) and financial difficulties (NRT vs. RT = 85.51 ± 16.89 vs. 62.16 ± 34.16, P = 0.002) were significantly different.

**Table 5:** Standard mean scores of quality of life (QoL) between two study groups.

	NRT (n = 23,)	RT (n = 74)	P
Global health status/QoL	72.10 ± 14.78	64.30 ± 16.35	0.044*
Function scales			
Physical function	71.30 ± 18.90	61.80 ± 19.28	0.043*
Role function	60.87 ± 22.25	55.86 ± 23.32	0.365
Emotional function	68.12 ± 16.98	59.12 ± 16.15	0.023*
Cognitive function	86.96 ± 12.26	87.61 ± 12.66	0.827
Social function	39.85 ± 16.47	37.16 ± 14.48	0.453
Symptom scales/items			
Fatigue	71.98 ± 14.55	70.42 ± 19.05	0.719
Nausea and vomiting	79.35 ± 24.60	73.65 ± 24.44	0.332
Pain	70.29 ± 27.96	73.42 ± 20.99	0.566
Dyspnea	85.51 ± 19.66	78.83 ± 23.78	0.225
Sleep disturbance	76.81 ± 27.40	83.33 ± 26.60	0.310
Appetite loss	82.61 ± 22.18	68.92 ± 30.88	0.052
Diarrhea	72.46 ± 23.89	52.70 ± 33.11	0.009**
Constipation	89.86 ± 15.68	82.88 ± 23.57	0.187
Financial difficulties	85.51 ± 16.89	62.16 ± 34.16	0.002**

^*^P < 0.05.

**P < 0.01.

## Discussion

The existing studies on the prognosis of cervical cancer have shown that positive postoperative pathological factors, such as DSI, LVSI, LN metastases, and parametrial and surgical margin involvement, could evidently worsen the prognosis^12–15^. Adjuvant RT should be considered after the primary surgery if pathologic risk factors are discovered^16–18^. In this study, LN metastases and adjuvant RT were found to be the most significant independent prognostic factors for young patients with cervical SCC in southwestern China. Interestingly, adjuvant RT did improve the prognosis of young patients with LN metastases. However, there were few significant differences in PFS and OS for young patients without LN metastases who underwent adjuvant RT. Our results demonstrated that adjuvant RT decreased the risk of disease progression and improved overall survival, compared to the scenario when no further treatment was conducted. However, little evidence was found that adjuvant RT might impact the prognosis for young patients without LN metastases. With the gradual improvement of surgical techniques and methods, complete radical surgery was conducted in standard scales without positive surgical margin, parametrium, and residue. This is likely to increase the PFS and OS for young cervical SCC patients without LN metastases, even no adjuvant RT was performed following the primary surgery. In addition, it remains unclear whether the benefits of conducting RT outweigh the risks^19^. This result prompted us to re-evaluate the risks and benefits of adjuvant RT for young patients with cervical SCC who had undergone the primary surgery.

Previous publications noted that severe side effects and life-threatening toxicity were observed in 6% of irradiated patients, compared to 2% in randomly selected patients who took no further treatment. In addition, 4% patients treated with radiation after radical surgery had grade 4 toxicity^17,20^. We examined the side effects and toxicity issues for young patients who were or were not treated with adjuvant RT following the primary surgery. In this study, different morbidities and severities of treatment-related side effects occurred in two treatment modalities. Patients in the RT group exhibited greater intestinal dysfunction, such as diarrhea,bloody stool, and Grade 1 to grade 3 toxicity of radioproctitis. The most critical factor contributing to intestinal dysfunction is that RT to pelvic cavity can easily cause telangiectasis of the rectum and damages to the blood vessels of rectal tissues, causing mucous membrane to be pale and fragile, and finally necrotic^21^. It is also reported that RT to pelvic cavity could result in other parameters associated with intestinal dysfunction, such as an imbalance of intestinal bacteria, bile salt and lactose dysabsorption, and altered intestinal peristalsis^22–25^. Additionally, higher morbidity of severe lower extremities edema, even in grade 4 toxicity, was found in the RT group. Previous studies have revealed that pelvic lymphadenectomy and nodal irradiation are two leading factors contributing to the development of the lower extremities edema in patients with gynecological malignancies^26,27^. Lymphatic fluid flow is obstructed primarily by radical surgery, and irradiation of adjacent nodal basins can damage the unhealed lymphatic channels and promote fibrosis of the lymph node and its surrounding tissues^28,29^. Therefore, adjuvant RT exacerbated the lower extremities edema following pelvic lymphadenectomy.

Sexual dysfunction has been found in almost half the patients with early-stage cervical cancer treated with surgery and RT^30^. Our results of the FSFI, which measured the sexual function for both study groups, were as expected. We did find differences in terms of lubrication, satisfaction, and dyspareunia between the two groups based on the postoperative therapeutic type. The direct injury during radical surgery causes a shortened vagina and pelvic neural dysfunction, possibly resulting in sexual dysfunction^31–33^. However, if the young patient did not receive adjuvant RT, the impairment of sexal function could be reversed since the bilateral salpingo-oophorectomy was not performed and the ovarian function remained intact. According to the literature, vaginal stenosis, premature ovarian failure, and descreased libido caused by pelvic RT often result in irreversible sexual dysfunction^33,34^.

The prolonged survival of cervical cancer by RT has drawed researchers’ attention to its impact on the patient’s QoL. A remarkable result in our study was that different postoperative adjuvant therapies caused significant differences in the long-term QoL. Some treatment-related symptoms did develop, as revealed by the QLQ-C30. Consistent with published studies, diarrhea worsened because of the toxicity of adjuvant RT^35,36^. Meanwhile, financial difficulties have been found to be a significant factor influencing young SCC patients’ QoL following adjuvant RT. According to our investigation and the literature, financial difficulties arose not only from work interruption, loss of employment and family income during the primary therapy, but also from the high medical expenses of RT and the lack of health insurance coverage in the developing areas of China^37,38^. Besides, patients in the RT group scored lower on function scales, including their physical and emotional functions. They did not recover or improve as time went by—this finding was largely inconsistent with those in previous studies^39,40^. Meanwhile, it is noteworthy that young patients who took adjuvant RT had worse long-term global QoL after five years. These results suggested that young patients with early-stage SCC who have pathologic risk factors following the primary surgery should be counseled in detail before the decision to use adjuvant RT can be made. The counseling should emphasize not only the benefit that local recurrence rates can be reduced, but also the risks that treatment-related side effects could increase and lower QoL could occur.

In addition, there were some limitations in this retrospective study. The clinician treatment modalities were uncontrolled, and non-random sampling may cause potential sampling bias. In addition, this retrospective study may underestimate both the frequency and severity of sequelae. These issues can be mitigated using random sampling or randomized experimental designs in the future.
